# Diet quality and anthropometric indices of patients undergone bariatric surgery: the prospective Tehran obesity treatment study

**DOI:** 10.1186/s12893-023-02032-4

**Published:** 2023-05-12

**Authors:** Firoozeh Hosseini-Esfahani, Mona Kazemi-Aliakbar, Glareh koochakpoor, Maryam Barzin, Alireza Khalaj, Majid Valizadeh, Parvin Mirmiran

**Affiliations:** 1 Nutrition and Endocrine Research Center, Research Institute for Endocrine Sciences, Shahid Beheshti University of Medical Sciences, Tehran, Iran; 2 Maragheh University of Medical Sciences, Maragheh, Iran; 3 Obesity Research Center, Research Institute for Endocrine Sciences, Shahid Beheshti University of Medical Sciences, Tehran, Iran; 4 Department of Surgery, Faculty of Medicine, Tehran Obesity Treatment Center, Shahed University, Tehran, Iran; 5 Department of Clinical Nutrition and Dietetics, Faculty of Nutrition and Food Technology, National Nutrition and Food Technology Research Institute, Shahid Beheshti University of Medical Sciences, Tehran, Iran

**Keywords:** Bariatric surgery, Sleeve gastrectomy, Gastric bypass, Dietary intake, Dietary quality, Obesity

## Abstract

**Background:**

Patients undergone bariatric surgery (BS) has long-term risks including decrease in diet quality, nutritional deficiencies and weight regain. This study focus on assessing dietary quality and food group components in patients one year after BS, the relationship between dietary quality score and anthropometric indices, and also evaluating the trend of body mass index (BMI) of these patients three years after BS.

**Methods:**

A total of 160 obese patients (BMI ≥ 35 kg/m^2^) were undergone sleeve gastrectomy (SG) (n = 108) or gastric bypass (GB) (n = 52), participated in this study. They were assessed for dietary intakes using three 24-hour dietary recalls one year after surgery. Dietary quality was assessed using food pyramid for post BS patients and healthy eating index (HEI). Anthropometric measurements were taken pre-surgery and 1, 2 and 3 years after operation.

**Results:**

The mean age of patients was 39.9 ± 11 years (79% female). The mean ± SD percentage of excess weight loss was 76.6 ± 21.0 one year after surgery. Intake patterns are generally (up to 60%) not consistent with the food pyramid. The mean total HEI score was 64 ± 12 out of 100. More than %60 of participants is exceeding the recommendations for saturated fat and sodium. The HEI score did not show significant relationship with anthropometric indices. The mean of BMI in SG group increased over three years of follow up, while in GB group, there were no significant differences in BMI during three years of follow up.

**Conclusions:**

These findings showed that patients had not healthy pattern intake one year after BS. Diet quality did not show significant relationship with anthropometric indices. The trend of BMI three years after surgery was different based on surgery types.

## Introduction

Overweight and obesity are defined as a condition that excess fat deposited in the body and increases risks for individual health care, especially chronic diseases [1]. These comorbidities include type 2 diabetes, hypertension, cardiovascular diseases (CVD), dyslipidemia, fatty liver disease, chronic kidney disease, obstructive sleep apnea, mood disorders and physical disabilities [2]. According to global research, obesity is a major public health epidemic in 21th century and data predicts increasing prevalence of overweight and obesity worldwide over the next years [3].

Bariatric surgery (BS) is the most useful treatment approach for classes II and III obesity, and has been associated with a decline in new incidence of CVD, cancer, type 2 diabetes, hypertension and mortality, as well as progression in quality of life [4]. Among several techniques of BS, Roux-en-Y gastric bypass (GB) and laparoscopic sleeve gastrectomy (SG) are the most frequently performed methods worldwide [5].

However, despite these benefits, without sufficient follow-up, BS has long-term risks including decreasing diet quality and nutritional deficiencies due to reducing bioavailability and malabsorption of nutrients and food intolerances. Also weight regain is common in patients who undertake this type of treatment and the outcome of BS may not be successful among individuals [6, 7]; therefore long-term follow up for identifying these risks of surgery is necessary to prevent recurrence of comorbidities related to obesity.

Energy-restricted diets, besides macronutrient imbalance and inadequate micronutrients can develop nutritional deficiencies, such as protein malnutrition, anemia and osteoporosis. Most obese patients have nutritional deficiencies prior to BS due to unbalanced diets; the anemia prevalence specially iron deficiency increases significantly after BS about 10–63% [8]. Calcium and vitamin D deficiencies are the two most important factors of sharpen bone loss after BS [9]. Calcium deficiencies are caused by a reduction in calcium absorption and low calcium intake due to an intolerance to milk products after BS [10]. Daily use of multivitamin and mineral supplements is usually recommended to prohibit post-surgical deficiencies [11]. Protein malnutrition is one of the most important problems occurred after operation, so it is recommended for bariatric patients that they keep an eye on their daily protein dose, 60–120 g (1.5 g/kg ideal body weight) [7]. Improving diet quality and nutrient adequacy of patients undergone BS is important for their health, maintaining weight loss and quality of life. There were only few studies expressing diet quality and long term consequences of weight in patients after BS. So this study focus on assessing dietary quality through food pyramid for post BS patients and healthy eating index (HEI) in patients one year after SG and GB surgeries, investigating the relationship between dietary quality score and anthropometric indices and also evaluate the trend of body mass index (BMI) of these patients three years after BS.

### Subjects

This study was performed within the frame of the Tehran Obesity Treatment Study (TOTS), an ongoing prospective study on severely obese subjects aged 18–65 years, receiving BS in an obesity treatment center. The BMI levels of participants were ≥ 40 (obesity class III) or 30 < BMI < 35 kg/m^2^ (obesity class II) with a medical comorbidity or failure of intensive medical and nutritional treatment for at least 1 year. They accepted surgical risk, provided an informed consent and guaranteed for regular follow-ups. More information considering the study protocol is available elsewhere [12]. Our care team post- surgery contained an obesity expert, nutritionist, and exercise medicine physician [12].

From March 2017 to March 2018, 383 obese patients (BMI ≥ 35 kg/m2) were undertaken BS by an experienced surgical team. Among the participants, those using anti-reflux drugs (n = 38) due to their interactions with vitamin B12 absorption, and patients who consuming corticosteroids (n = 15) due to their side effects, such as weight gain and muscle weakness, those with gastrointestinal complications related to surgery (n = 42), and subjects with incomplete 24-hour dietary recalls 12 months after surgery (n = 128) were excluded. Finally, 160 (female = 128, male = 34) individuals entered the study. All of the participants had undergone BS (SG = 108 or GB = 52) and were assessed for dietary intakes and anthropometric outcomes one year post surgery. Information regarding long term weight regains or weight loss is provided from annual follow up meetings 3 years after the surgery through anthropometric measurements. Patients were undertaken nutritional counselling and administered dietary guidance annually.

Patients undertook bariatric procedures via laparoscopy by a single surgical team under general anesthesia. The mainly type of surgery was SG, which was performed using a 36-F bougie with excision of 80% of the stomach. On the other hand, Roux-en-Y GB was conducted using an alimentary limb of 100–150 cm and a biliopancreatic limb of 50 cm or minigastric bypass (loop gastroenterostomy of 160 cm) [12].

The ethics Committee of the Research Institute for Endocrine Sciences, Shahid Beheshti University of Medical Sciences, Tehran, Iran (IR.SBMU.ENDOCRINE.REC.1395.203) approved all methods about caring human participants. This study was performed in accordance with the ethical standards of Declaration of Helsinki (1964) and its later comparable ethical standards. All of the participants included in the study completed written informed consents.

## Materials and methods

### Measurements

The patients were visited in accordance with the standard protocol at 1, 3, 6, 12, 24 and 36 months after surgery as fallow ups. Anthropometric indices were taken pre-surgery and 1, 2 and 3 years after operation. BMI was determined by dividing weight in kilograms by height in squared meters. The waist circumference (WC) was measured at the mid-point between the lowest rib and the iliac crest to the nearest 0.5 cm. Also the body fat percentage and fat free mass was measured by the body composition analyzer (InBody 370, Biospace, Seoul, Korea). Weight loss was equaled as change in BMI and percentage of excess weight loss (EWL%), with ideal weight defined as BMI of 25 kg/m^2^.

### Nutritional assessment

A calorie-restricted diet according to the age, sex, and daily activity level of the patients was prescribed by a trained dietician in the follow-up visits. The energy balance was negative, and on the first days after surgery daily calorie intake ranged from 400 to 500 kcal and it is promoted to about 1000 kcal at the end of the first year; protein intake should not be less than 60 g/day [13].

For up to 12 months, our experts were prescribed daily vitamin and mineral supplements for all patients. The post-surgery oral multivitamin/mineral supplements consisted one Pharmaton® capsule (containing 2 mg of copper, 10 mg of ferrous sulfate, 100 mg of folic acid, and 1mcg of vitamin B12, vitamin A, vitamin B group, vitamin C, vitamin D, vitamin E, nicotinamide, and biotin; Boehringer Ingelheim Inc., Germany) and one Calcicare tablet (200 IU of vitamin D, 400 mg of calcium, 100 mg of magnesium, and 4 mg of zinc) daily. The patients were prescribed and guided for dietary plan and physical activity based on their individual assessments.

A skilled dietician group assessed nutritional intakes of participants 12 months after operation. The three 24-h dietary recalls was filled for 2 non-consecutive weekdays and 1 day of the weekend. We measured each food item intake per day (grams/day) using local household measures. The US Department of Agriculture [14] and Iranian Food Composition Table [15] were used to convert crude data to grams, milligrams or micrograms of nutrients.

The average of three days dietary intake assessments were calculated. Dietary intakes were compared with food pyramid for post BS patients. The base of food pyramid emphasizes physical activity, vitamin and mineral supplementations. High protein foods with low fat content are recommended at the first level of pyramid. The second level consists of fruit, vegetable and oil. The third and fourth levels of pyramid respectively recommend whole grains and limit sweets and energy dense foods including cakes, candies, fatty meats, butter and cream [16].

Also newer dietary pattern screening tool like HEI has been associated with health outcomes, represents variation in diet quality independent of diet quantity and considers the multidimensional nature of healthy diets. Specifically it is useful for measuring dietary components that are not considered in food pyramid for post BS patients [17].

The HEI score (13 dietary integrals) is based on recommendations of the 2015–2020 dietary guidelines for Americans. A score for each dietary component is determined by calculating the ratio of component intake to the standard intake. The score of each component was summed to estimate the total HEI score, ranging from 0 to100. The patient who had higher total HEI score had greater adherence to dietary guideline recommendations. Nine adequacy components (total fruits, whole fruits, total vegetables, greens and beans, whole grains, dairy, total protein foods, seafood and plant proteins, and fatty acids) and four moderation components (those are more unhealthy to be limited) including refined grain, sodium, added sugars, and saturated fats. The HEI score depends on density; therefore, dieticians computed the ratio of fatty acids and the amount of dietary integrals per 1000 kcal of energy intake. Recommendations are in the range of 1200–2400 kcal. Six of adequacy components, including total fruit, whole fruit, total vegetables, greens and beans, total protein foods, seafood and plant proteins, each gave a score of 0 and 5 for the lowest and highest intake, respectively. The other three adequacy components consist of whole grains, dairy and fatty acids were ranged from 0 to 10 for the lowest to highest intake, respectively. The four moderation components including refined grains, sodium, added sugars, and saturated fats (SFA), acquired a score of 0 and 10, respectively for the highest and lowest intakes. The intermediate intake score were considered between the minimum and maximum score [18]. Also food group intakes were assessed based on weekly or daily recommended amounts of food group intakes for a 1000 kcal diet of dietary guidelines for Americans 2020–2025 [19].

### Statistical analysis

For all statistical analyses, SPSS version 20 (IBM Corp., Chicago, USA) was applied. Continuous variables are shown as mean ± standard deviation (SD), and categorical variables are displayed as frequency (percentage). To compare pre- and post- surgery of anthropometric variables, paired t-test was used. Linear regression analysis adjusted by pre-surgery weight was performed for determining the association between HEI score, its components and anthropometric outcomes in patients one year after surgery. The generalized estimated equation (GEE) method was used to evaluate time trends of BMI for up to 3 years after BS. The model were fitted for confounding factors including sex, age, pre-surgery BMI, time, surgery type (SG or GB), total score HEI and the interaction of time and surgery type. The marginal means in each year and P for interaction were computed based on GEE model (P < 0.05), so the model was done based on two surgery types. The correlation between repeated measurements considered in the GEE model.

## Results

The characteristics of the study population (preoperative and postoperative) are shown in Table 1. The mean age of patients was 39.9 ± 11 years. About 79% of participants were female. There were significant differences in terms of all anthropometrical indices between pre- and post-surgery. The mean percentage of excess weight loss was 76.6 ± 21.0%.

**Table 1:** Characteristics of the participants undergone bariatric surgery (n = 160)

Characteristics	Pre-operative	Post-operative (one year)	P value
Age (years)		39.9 ± 11.5	
Sex (female %)		78.8%	
Height (cm)		162 ± 9.45	
Weight (kg)	119 ± 20.0	80.3 ± 15.4	< 0.001
BMI (kg/m^2^)	45.1 ± 6.46	30.4 ± 5.29	< 0.001
WC (cm)	125 ± 13.6	96.4 ± 12.2	< 0.001
Hip circumference	135 ± 12.5	108 ± 11.8	< 0.001
WC/Height ratio	0.93 ± 0.09	0.88 ± 0.08	< 0.001
Marital status, married (%)		70.3%	
Education > 12		42.7%	
Smokers (%)		12.5%	
Alcohol consumption (%)		14.4%	
Fat mass (kg)	59.0 ± 11.9	28.7 ± 10.4	< 0.001
Fat free mass (kg)	58.8 ± 11.0	50.6 ± 10.0	< 0.001
Lean mass (kg)	54.7 ± 8.88	47.3 ± 9.64	< 0.001
Weight loss (kg)		-38.9 ± 12.3	
BMI loss (kg/m^2^)		-14.7 ± 4.34	
Excess weight loss (%)		76.6 ± 21.0	

BMI: Body mass index, WC: Waist circumference

Values are mean ± SD unless otherwise listed

Paired t-test was used to compare pre- and post- surgery of anthropometric variables

Table 2 presents the average food group intakes one year after surgery compared with nutritional pyramid for post-gastric bypass patients. Intake patterns are generally (up to 60%) not consistent with the healthy dietary guidelines. Average intakes of protein foods, vegetable, fruits and oils fall below the range of recommended intakes. Mean consumption of grains exceed the upper end of the recommended intake. Current patterns of protein foods including seafood, dairy, legumes and egg were below one serving/day consumption.

**Table 2:** Food group intakes one year after surgery compared with nutritional pyramid for post-gastric bypass patients

Food groups	Dietary intakes (gr/day)	Dietary intakes (Serving)	% of inadequacy	Recommended intakes/day
Total energy (kcal/day)	1220 ± 690			
Protein foods	160 ± 103	3.60 ± 1.85	60.6	4–6
Meat (red meat, chicken)	62.7 ± 45.9	2.15 ± 1.57		
Seafood	5.34 ± 13.5	0.18 ± 0.48		
Dairy (cheese, milk, yoghurt)	64.3 ± 82.9	0.55 ± 0.49		
Legumes	11.3 ± 25.4	0.12 ± 0.27		
Eggs	16.6 ± 19.4	0.58 ± 0.68		
Fruit (low and high sugar fresh fruit)	132 ± 110	0.83 ± 0.69	82.4	2–3
Vegetable	72.7 ± 68.7	0.54 ± 0.53	70.6	2–3
Grains (rice, pasta, bread, breakfast cereals, legumes)	132 ± 83.1	2.90 ± 1.95	73.1	2
Oil (olive oil, sunflower oil)	6.08 ± 6.49	0.05 ± 0.13	100	2–3

Food group intakes were assessed based on daily recommended amounts of nutritional pyramid for post-gastric bypass patients

Table 3 presented the total HEI score and its components, which is a measure of how intakes align with the Dietary Guidelines. The total HEI score was 64 ± 12 out of 100 in patients 1 year after surgery. More than %60 of participants is exceeding the recommendations for saturated fat and sodium, while only %31 of participants are consuming added sugars upper than healthy pattern. Linear regression analysis of the association between total HEI score and its components with postoperative anthropometric indices is presented in Table 4. The HEI score did not show significant relationship with anthropometric indices. Added sugar score positively associated with postoperative waist circumference (P < 0.05). Seafood score index positively associated with postoperative lean mass (P < 0.05). The other components of HEI score had no relationship with anthropometric indices.

**Table 3:** Healthy eating index (HEI) scores of participants one year after surgery

Component	Scores mean ± SD	Inadequacy* n (%)	Max points
**Adequacy items**			
Total fruits	3.61 ± 1.73	85 (53.1)	5
Whole fruits	4.81 ± 1.61	45 (28.1)	5
Total vegetables	2.70 ± 1.84	119 (74.4)	5
Greens and beans	2.52 ± 2.15	104 (65)	5
Whole grains	2.87 ± 3.50	142 (88.8)	10
dairy	4.25 ± 3.10	146 (91.3)	10
Total protein foods	3.94 ± 1.41	77 (48.1)	5
Seafood and plant proteins	1.38 ± 1.85	141 (88.1)	5
Fatty acid ratio†	7.18 ± 3.53	93 (58.1)	10
**Moderation items**			
Refined grains (serving/1000 Kcal/day)	7.81 ± 3.49	66 (41.3)	10
Sodium (mg/1000 kcal/day)	6.85 ± 3.52	98 (61.3)	10
Added sugars (% of energy)	9.29 ± 1.64	50 (31.3)	10
Saturated fat (% of energy)	7.25 ± 3.23	100 (62.5)	10
Total HEI score	64.0 ± 12.4		100

* Subjects who did not take full score in each food group

† (monounsaturated fat + polyunsaturated fat) / saturated fat, (% of energy)

**Table 4:** Linear regression analysis of the association between healthy eating index and its component scores with anthropometric outcomes of participants, one-year after bariatric surgery

	β	P value
Healthy eating index score		
Excess BMI loss	-0.005 ± 0.13	0.96
Postoperative lean mass	0.07 ± 0.06	0.28
Postoperative fat free mass	0.001 ± 0.05	0.99
Postoperative fat mass	-0.03 ± 0.06	0.68
Postoperative waist circumference	0.09 ± 0.07	0.23
**Total fruits score**		
Excess BMI loss	0.06 ± 0.91	0.94
Postoperative lean mass	0.007 ± 0.54	0.98
Postoperative fat free mass	-0.34 ± 0.38	0.37
Postoperative fat mass	0.18 ± 0.49	0.71
Postoperative waist circumference	0.91 ± 0.56	0.10
**Whole fruit score**		
Excess BMI loss	-0.20 ± 0.98	0.84
Postoperative lean mass	-0.16 ± 0.65	0.80
Postoperative fat free mass	-0.47 ± 0.42	0.26
Postoperative fat mass	0.24 ± 0.54	0.66
Postoperative waist circumference	0.87 ± 0.62	0.16
**Total vegetable score**		
Excess BMI loss	0.36 ± 0.85	0.67
Postoperative lean mass	0.05 ± 0.45	0.91
Postoperative fat free mass	0.34 ± 0.35	0.34
Postoperative fat mass	0.11 ± 0.44	0.80
Postoperative waist circumference	0.05 ± 0.51	0.92
**Greens and beans score**		
Excess BMI loss	0.42 ± 0.73	0.56
Postoperative lean mass	0.33 ± 0.35	0.36
Postoperative fat free mass	-0.07 ± 0.29	0.82
Postoperative fat mass	-0.07 ± 0.37	0.85
Postoperative waist circumference	-0.12 ± 0.37	0.74
**Whole grain score**		
Excess BMI loss	0.008 ± 0.45	0.98
Postoperative lean mass	0.24 ± 0.20	0.26
Postoperative fat free mass	0.18 ± 0.18	0.31
Postoperative fat mass	-0.25 ± 0.23	0.28
Postoperative waist circumference	-0.06 ± 0.26	0.81
**Dairy score**		
Excess BMI loss	0.06 ± 0.51	0.90
Postoperative lean mass	0.15 ± 0.24	0.54
Postoperative fat free mass	0.28 ± 0.42	0.51
Postoperative fat mass	-0.18 ± 0.26	0.47
Postoperative waist circumference	0.18 ± 0.30	0.55
**Total protein score**		
Excess BMI loss	0.86 ± 1.11	0.44
Postoperative lean mass	0.87 ± 0.64	0.17
Postoperative fat free mass	0.71 ± 0.47	0.13
Postoperative fat mass	-0.98 ± 0.60	0.10
Postoperative waist circumference	0.40 ± 0.70	0.57
**Seafood score**		
Excess BMI loss	-0.03 ± 0.86	0.97
Postoperative lean mass	0.83 ± 0.38	0.03
Postoperative fat free mass	0.46 ± 0.34	0.17
Postoperative fat mass	-0.07 ± 0.43	0.86
Postoperative waist circumference	0.61 ± 0.50	0.22
**Refined grain score**		
Excess BMI loss	0.03 ± 0.45	0.94
Postoperative lean mass	-0.10 ± 0.18	0.58
Postoperative fat free mass	-0.03 ± 0.17	0.85
Postoperative fat mass	0.02 ± 0.22	0.94
Postoperative waist circumference	-0.02 ± 0.22	0.91
**Sodium score**		
Excess BMI loss	-0.15 ± 0.45	0.73
Postoperative lean mass	0.07 ± 0.23	0.75
Postoperative fat free mass	0.21 ± 0.17	0.24
Postoperative fat mass	-0.04 ± 0.22	0.84
Postoperative waist circumference	0.04 ± 0.22	0.86
**Added sugar score**		
Excess BMI loss	-0.69 ± 0.97	0.48
Postoperative lean mass	-0.48 ± 0.45	0.30
Postoperative fat free mass	-0.25 ± 0.43	0.56
Postoperative fat mass	0.62 ± 0.54	0.25
Postoperative waist circumference	1.46 ± 0.62	0.02
**Fatty acids score**		
Excess BMI loss	0.16 ± 0.45	0.71
Postoperative lean mass	0.16 ± 0.27	0.56
Postoperative fat free mass	-0.03 ± 0.18	0.85
Postoperative fat mass	-0.12 ± 0.23	0.60
Postoperative waist circumference	-0.06 ± 0.26	0.83
**Saturated fatty acid score**		
Excess BMI loss	-0.23 ± 0.49	0.63
Postoperative lean mass	0.14 ± 0.26	0.60
Postoperative fat free mass	-0.16 ± 0.20	0.41
Postoperative fat mass	0.05 ± 0.25	0.84
Postoperative waist circumference	0.01 ± 0.29	0.97

The trend of body mass index (BMI) over three years of follow-up based on surgery type

Roux-en-Y gastric bypass (GB)

Laparoscopic sleeve gastrectomy (SG)

**Fig. 1:**
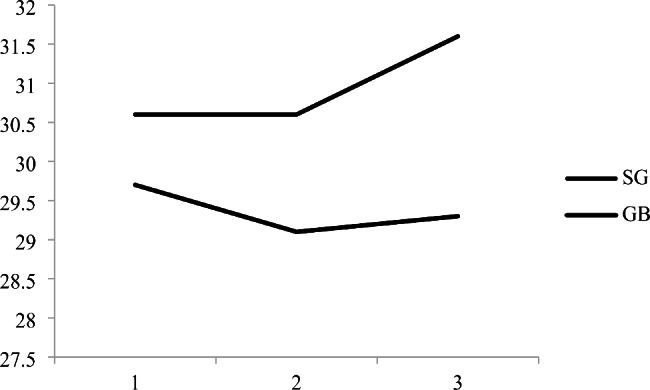
The trend of body mass index (BMI) over three years of follow-up based on surgery type Roux-en-Y gastric bypass (GB) Laparoscopic sleeve gastrectomy (SG)

Follow up rates at 1, 2 and 3 years after surgery were respectively 100, 65 and 64%. There was an interaction term between follow up time and surgical technique groups (SG and GB) (P interaction < 0.05) after controlling for confounding variables including sex, age, pre-surgery BMI, time, surgery type (SG or GB) and total score HEI. The means of BMI in SG group increased over three years of follow up, while in GB group, there were no significant differences in BMI during three years of follow up (Fig. 1).

## Discussion

Three major findings were reveals in our study. First, one year after BS, more than 60% of BS patients had unfavorable diet quality based on two criteria of food pyramid for BS patients and HEI score, this issue was more significant for food groups including oil, fruit, vegetable, whole grain and protein foods. Second, no significant association was observed between HEI score and anthropometric parameters; however, there were positive relationships between added sugar score and postoperative WC and seafood score with postoperative lean mass. Third, in SG group the mean of BMI increased over three years of follow up, but this increase does not occur in the GB group.

Few studies have been conducted in the field of examining dietary quality in BS patients, and this limited amount has adopted a reduction approach. Previous studies more focused on monitoring nutrient intakes, food group intakes, or other dietary components [11, 20, 21]. There are inter-relationships in the whole diet; the synergistic or antagonistic effects of foods make it difficult to separate the effect of single food or nutrients, so examining it as a whole (dietary pattern) certainly helps to predict the relationship between diet and disease outcomes accurately. Also nutrition recommendations in the framework of food groups are easier for people to understand than the concept of nutrients [22].

As far as we know, our study is the first that investigated the quality of whole diet of BS patients using two indices. The poor diet quality of the BS patients is the main finding of the present study. In our study, the mean intake of whole grains and vegetables particularly fell below the range of recommended intakes. Similarity, In Zekiye Erdem et al. study [23], in which the quality of diet were examined compared with bariatric food pyramid recommendations, consumption of vegetable, fruit and vegetable oil were below the recommended amount. In a cross-sectional study by Jabbour and et al. [4] most patients consumed less vegetables one to three years after surgery compared to pre-surgery diet. Also previous study reported that a low proportion of BS patients consumed adequate amounts of foods from fruit, vegetable, protein and vegetable oil food groups and they consumed high carbohydrates, sugars and fats one year post-surgery [24].

Based on the available guidelines, bariatric patients are advised to limit fiber-rich foods such as fruits, vegetables and whole grains during the first 4 months after surgery; however, after this time, patients are allowed to reintroduce all foods to their diet, but it seems that not consuming these foods for four months makes it difficult for the patient to consume them again, and these foods are not successfully reintroduced to the patients diet [13].

In the present study, higher HEI score were not significantly associated with postoperative anthropometric indices. Previous study reported that weight regain was not associated with HEI before or after surgery [4]. Previous evidences confirmed that adherence to Mediterranean diet may be related to weight loss before or after surgery [25]. The lack of significant association with BMI or postoperative body composition might be explained due to several reasons. In this study few subjects had high HEI scores and most subjects had low scores for the components that were potentially associated with BMI reduction, i.e., vegetables and whole grain. The mean HEI score observed in our study were around 64.3, whereas the mean HEI scores in studies that reported a positive association between weight loss and HEI score in general population are more than 64 [26]. Energy intake and physical activity levels before surgery are effective on postoperative weight loss [27]; however, the pre-surgery dietary quality and the level of physical activity of BS patients were not available in this study [28].

In our study, only %31 of participants are consuming added sugars upper than the healthy dietary pattern. Therefore, the interpretation of the observed relationships for these subjects does not seem logical and requires further studies. The standards for maximum and minimum level of score [29] may be adapted for BS patients and based on low energy level intakes.

Based on our result, patients undergoing SG had the higher weight regain in comparison with GB patients. Some BS patients regain most of all weight loss over time. Previous studies showed that weight regain after BS were associated with diabetes progression and decline in quality of life [21], so the results of this study indicating weight regain may be clinically important and should be noticed. This is similar to findings of other studies [30–33]; in study by Himpens et al. [31] weight regain was appeared in 75.6% of SG patients after the sixth post-operative year. In this study, the weight gain of patients who received only SG was higher than that of patients who also had duodenal switch in addition to SG, so it seems that long-term outcome of obesity surgery depend on techniques performed. There is evidence that after SG, even after performing a narrow gastric tubulization, the gastric capacity can increases, so the final residual gastric volume will determine the late clinical results of SG [34]. Eating behaviors including increased portion size and sweet intakes may be dietary risk factors for weight regain [35]. Previous meta-analysis reported that individuals with weight regain had higher energy (192 Kcal) and carbohydrate (25 gram) intakes than non-weight regains [21]; also physical activity helps maintain weight loss and make better body composition [24]; however, we had not dietary and physical activity data 2–3 years after surgery. Previous studies reported that the follow up rate of BS patients was 40–62% [36]. Our finding confirmed this challenge. In the later stages of BS (two to three years later) weight regain are noticed, because the effect of BS decreased and patients re-adopt unhealthy behaviors [37], so the interactive contact of multidisciplinary care team with BS patients over the long term is necessary to promote healthy lifestyle skills and manage body weight.

There are several strengths and limitations in our study. The prospective design is the main strength of our study. Other strengths of our study include assessing dietary intake of participants by expert dietitians. Extracting priori-dietary patterns in the form of HEI or food pyramid make it easier to compare with other studies. The main limitation in this study was evaluating food intake only once, one year after surgery, while it would have been better to monitor food intake before surgery and two to three years after surgery. The majority of participants in this study were females, which may cause a selection bias. Also we had no information about the compliance of patients on prescribed daily vitamin and mineral supplements and physical activity.

## Conclusion

Based on two measures of dietary quality, food pyramid for BS patients and HEI, more than 60% of the BS patients had suboptimal diet quality for majority of food groups (especially for oil, fruit, vegetable, whole grain and protein foods). The means of BMI in SG group increased over three years of follow up, but this increase does not occur in GB group.

## Data Availability

The datasets used and/or analyzed during the current study are available from the corresponding author on reasonable request.
